# Long-Term Physical Aging Tracked by Advanced Thermal Analysis of Poly(*N*-Isopropylacrylamide): A Smart Polymer for Drug Delivery System

**DOI:** 10.3390/molecules25173810

**Published:** 2020-08-21

**Authors:** Anna Czerniecka-Kubicka, Iwona Zarzyka, Marek Pyda

**Affiliations:** 1 Department of Experimental and Clinical Pharmacology, Medical College of Rzeszow University, The University of Rzeszow, 35-310 Rzeszow, Poland; 2 Department of Chemistry, Rzeszow University of Technology, 35-959 Rzeszow, Poland; izarzyka@prz.edu.pl (I.Z.); mpyda@utk.edu (M.P.); 3 Department of Biophysics, Poznan University of Medical Sciences, 60-780 Poznan, Poland

**Keywords:** poly(*N*-isopropylacrylamide), enthalpy relaxation, physical aging, advanced thermal analysis, equilibrium enthalpy

## Abstract

Poly(*N*-isopropylacrylamide) (PNIPA), as a smart polymer, can be applied for drug delivery systems. This amorphous polymer can be exposed on a structural recovery process during the storage and transport of medicaments. For the physical aging times up to one year, the structural recovery for PNIPA was studied by advanced thermal analysis. The structural recovery process occurred during the storage of amorphous PNIPA below glass transition and could be monitored by the differential scanning calorimetry (DSC). The enthalpy relaxation (recovery) was observed as overshoot in change heat capacity at the glass transition region in the DSC during heating scan. The physical aging of PNIPA was studied isothermally at 400.15 K and also in the non-isothermal conditions. For the first time, the structural recovery process was analyzed in reference to absolute heat capacity and integral enthalpy in frame of their equilibrium solid and liquid PNIPA.

## 1. Introduction

**Figure 1:**
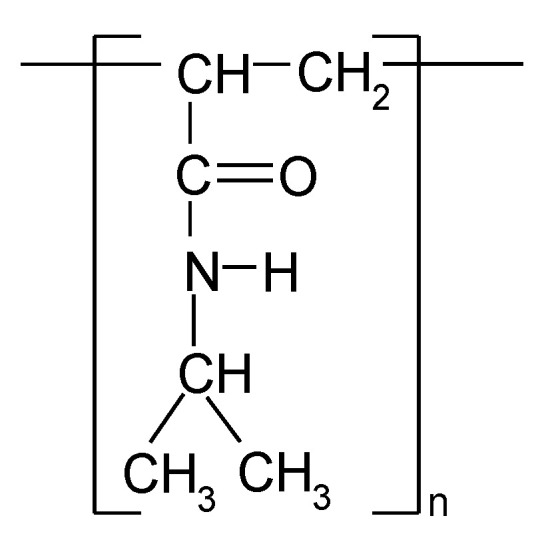
Chemical structure of the repeating unit of poly(*N*-isopropylacrylamide) (PNIPA).

Poly(*N*-isopropylacrylamide) (PNIPA) belongs to the smart polymer group (Figure 1). It is an ideal candidate for use in drug delivery systems because it is a biocompatible, a low-toxic, and has a Lower Critical Solution Temperature (LCST) around human body temperature. PNIPA is a thermoresponsive polymer having LCST = 32 °C. Above the LCST, the PNIPA is hydrophobic and interacts with the components of the cells, while, below that, it is hydrophilic and does not interact with them [1,2,3,4,5]. The extensive research has been carried out on utilizing this property of PNIPA for the delivery of drugs in stimuli responsive drug delivery systems [1,2,3,4,5,6,7,8,9,10,11,12,13,14,15,16,17]. These properties of PNIPA have caused this thermosensitive polymer to be utilized in many drug delivery systems, including for cancer therapeutics. In response to changes in temperature, solutions of PNIPA exhibit rapid, reversible phase transition/phase separation phenomena.

**Figure 2:**
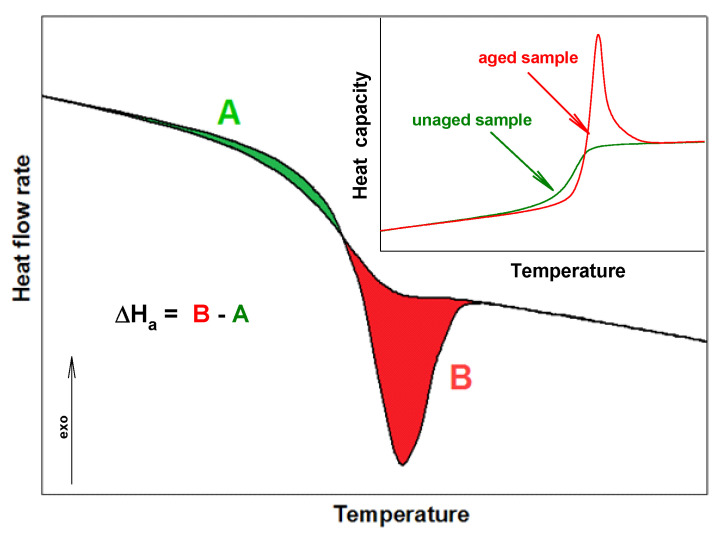
Scheme of the enthalpy relaxation estimation from standard differential scanning calorimetry (DSC) measurements.

PNIPA is an amorphous polymer which easily ages. This process, commonly so-called a structural recovery, is linked with storage of material. If the storage temperature is below a temperature of glass transition (T_g_), the structural recovery (physical aging) of an amorphous or a semicrystalline substance is observed. This process applies to natural and synthetic materials. The structural recovery can be monitored by the volume recovery or the enthalpy recovery [18,19,20,21,22,23,24,25,26]. In literature, the physical aging term is also used, but it applies to changes in mechanical properties. The result of the physical aging process is the change of material properties, such as in length, hardness, and brittleness [23,24,25,26,27,28,29,30]. On the other hand, the physical properties of amorphous and semicrystalline material at the non-equilibrium state are changed versus time and temperature. In the case of the amorphous material, the values of volume, enthalpy, and entropy are higher than the value for material at the equilibrium state. Thermodynamic properties at the storage temperature below T_g_ are changed in the direction of equilibrium value. It is linked with the ordering of molecules under process conditions [31,32,33,34]. The characteristic thermogram of the aging process in the differential scanning calorimetry (DSC) measurement shows overlapping the enthalpy relaxation on the change of heat capacity in the glass transition region, as is presented in Figure 2 [34,35,36,37,38].

Enthalpy relaxation (ΔH_a_) and the fictive temperature (T_f_) are parameters which describe the progress of structural recovery in DSC experiments [38,39,40,41,42,43]. The enthalpy relaxation can be estimated as a difference between the A and B areas (see Figure 2). These areas are bounded of aged and unaged curves of heat flow rate or heat capacity for these full amorphous or semicrystalline samples [43,44,45]. The formation of ΔH_a_ results from the aim for the amorphous material to state at better thermodynamic stability, and T_f_ is the second parameter related to the structural recovery, which characterizes the structure of the amorphous phase. The fictive temperature can be determined directly based on the DSC measurement during the heating process by analyzing the raw data of aged and unaged heat flow rate (or heat capacity) or based on analysis of total enthalpy function [46,47,48,49,50,51,52,53].

**Figure 3:**
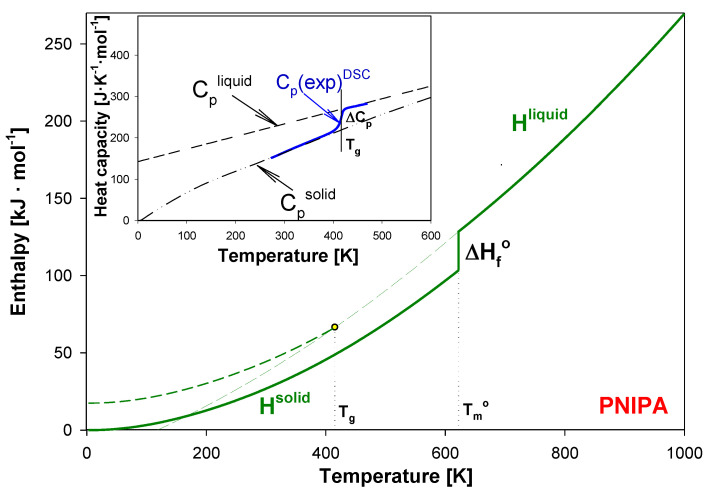
Enthalpy of liquid (H^liquid^) and solid (H^solid^) of poly(*N*-isopropylacrylamide) as the temperature function according to [54]. In the insert, the experimental heat capacity of poly(*N*-isopropylacrylamide) is presented in reference to equilibrium solid, C_p_^solid^, and liquid, C_p_^liquid^, heat capacities [54].

The scheme presented in Figure 3 shows the quantitative analysis of the enthalpy relaxation estimation. This approach established and used the equilibrium solid, C_p_^solid^, and liquid, C_p_^liquid^, heat capacities as reference lines.

The baselines at solid (C_p_^solid^) and liquid (C_p_^liquid^) state of PNIPA were established early in Ref. [54]. The insert of Figure 3 shows an example of the advanced thermal analysis for experimental heat capacities, C_p_(exp)^DSC^ obtained from the standard DSC, using C_p_^solid^ and C_p_^liquid^ heat capacities. The advanced thermal analysis of glass transition using the equilibrium solid and liquid heat capacity allowed to establish the glass transition temperature (T_g_) with estimated value of 415 K and the change of heat capacity (ΔC_p_) of PNIPA at T_g_ as 50.36 J·mol^−1^·K^−1^ [54].

In this paper, for a first time, the structural recovery of PNIPA is monitored based on the experimental thermodynamic functions enthalpy, and transition parameters of PNIPA referenced to equilibrium enthalpy of solid (H^solid^) and liquid (H^liquid^) [45,54]. Figure 3 shows the H^solid^ and H^liquid^ of PNIPA as the temperature function. At equilibrium melting temperature of 622 K, the value of H^solid^ increased by the value of the equilibrium heat of fusion, ∆H_f_°, of 26.591 kJ·mol^−1^ to reach the level of H^liquid^ [45,54].

**Figure 4:**
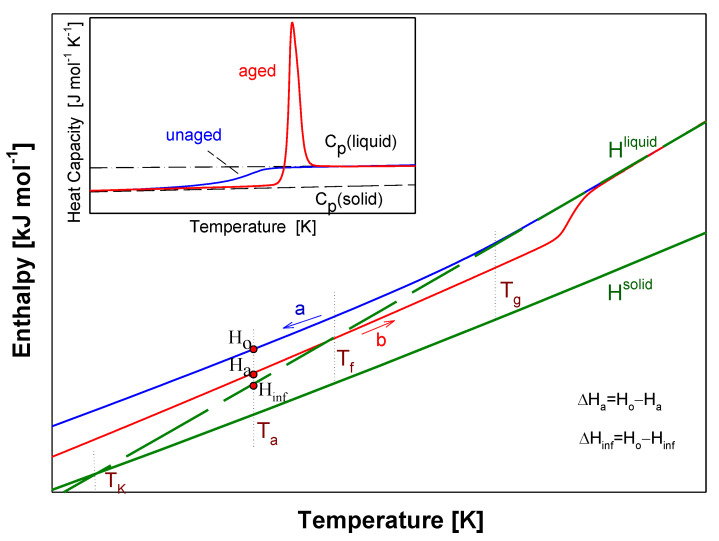
Scheme of the analysis of experimental enthalpy recovery of polymeric material in the frame of equilibrium enthalpies (H^solid^, H^liquid^) versus temperature. The insert shows the aged and unaged, experimental heat capacity carried out by the differential scanning calorimetry (DSC) measurement. The solid, C_p_(solid), and liquid, C_p_(liquid), heat capacity are presented as equilibrium lines of references.

Figure 4 shows a scheme of the results from advanced thermal analysis of physical aging of full amorphous polymeric material in details at around glass transition temperature (T_g_) similar as in Ref [45]. First, the insert of Figure 4 illustrates the experimental apparent heat capacities of both unaged and aged samples in frame of equilibrium solid C_p_(solid) and liquid C_p_(liquid) heat capacities. The unaged sample presents a step in heat capacity at T_g_ and aged sample shows an endothermic peak. Figure 4 primarily shows the integral enthalpy function of aged (curve b) and unaged (curve a) polymer versus temperature compared with equilibrium liquid H^liquid^ and solid H^solid^ enthalpies for data C_p_ presented in the insert this figure. The physical aging process described in Figure 4 was carried out isothermally at the aging temperature (T_a_).

The H^liquid^ line has been elongated towards the low temperatures (dash line). Under cooling the polymeric material at liquid state at a given rate, the enthalpy of aged polymer diffracted at the glass transition temperature (T_g_). The unaged material reached the H_o_ value at T_a_ and then, subjected to the isothermal aging process, lowered its enthalpy to the H_a_ value at the aging temperature (T_a_). The curve ‘a’ indicates the enthalpy of unaged polymer obtained during the cooling, while the curve ‘b’ points at the enthalpy of the aged polymer obtained under the heating. The intersection of the experimental enthalpy (the curve ‘b’) of the aged polymer with the extension of H^liquid^ determines the so-called fictive temperature (T_f_) [47,48]. If the extrapolated liquid line is not reached at the completion of structural recovery, the fictive temperature should cease to evolve when T_f_ = T_a_. However, several studies of enthalpy recovery reported that the extrapolated liquid line is not reached even at the completion of enthalpy recovery [27,41,42,55,56,57,58,59]. The experimental H_a_ values reached the H^liquid^ level more above the T_g_ temperature which is the result of the relaxation enthalpy peak.

In Figure 4, the difference of H_a_-H_o_ equals the enthalpy recovery ∆H_a_ can be estimated from such calorimetric measurements. The temperature marked as T_K_ is so-called the Kauzman temperature (equilibrium glass transition temperature) [60,61,62,63]. The equilibrium enthalpy recovery is usually labeled as ΔH_inf_ and indicates the maximum value of enthalpy in an infinite aging time and is determined by the following equation:

(1) $$∆H_{\inf}=\int_{T_{a}}^{T_{g}}∆C_{p}\mathrm{dT}\approx ∆C_{p}\left(T_{g}-T_{a}\right),$$

where T_g_ is the glass transition temperature, ΔC_p_-the change of heat capacity between glassy and liquid states at T_g_, and T_a_ is the aging temperature [27,45]. The right part of Equation (1) is an approximated expression for estimation of ΔH_inf_, whereas the left part is the more advanced approach to one. In this second case, it is necessary to know C_p_(solid) and C_p_(liquid) as a function of temperature.

The progress of the kinetic in physical aging process can be also described by the estimation of the recovery parameter, φ. This parameter estimates a level of the relaxation material advancement in the reference to equilibrium state. The recovery parameter can be expression by following equation:

(2) $$\varphi =\frac{∆H_{\inf}-∆H_{a}}{∆H_{\inf}},$$

where ∆H_inf_ is an equilibrium enthalpy recovery, and ∆H_a_ is an experimental enthalpy recovery obtained at the aging temperature, T_a_, after the aging time, t_a_. The recovery parameter φ = 1 in the case of unaged sample, whereas φ = 0 if the ∆H_a_ achieves the value of equilibrium, ∆H_inf_ in the infinite time.

In this work, the dependence of enthalpy recovery and integral enthalpy on temperature was used to the advanced, quantitative thermal analysis of the physical aging process of PNIPA at isothermal conditions. Based on references of H^solid^ and H^liquid^ of PNIPA, thermal analysis was carried out and enthalpy recovery was estimated for the given aging time and for the equilibrium enthalpy recovery. The kinetic features of structural recovery at the glass transition region was described using the Kohlrausch–Williams–Watts (KWW) model [49,64,65].

## 2. Results and Discussion

### 2.1. Isothermal Aging of PNIPA

**Figure 5:**
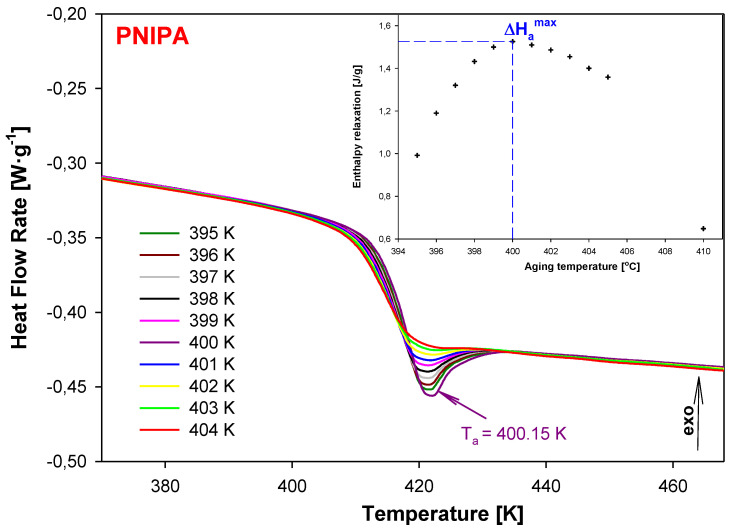
The dependence of heat flow rate on temperature obtained after the isothermal aging of PNIPA at different aging temperatures in the range of 395–404 K for 30 min. The maximum enthalpy recovery was obtained at T_a_ = 400.15 K. The insert shows the enthalpy recovery versus aging temperature.

Figure 5 shows the heat flow rate versus temperature of the structural recovery process for PNIPA carried out in the isothermal conditions for different aging temperatures (T_a_) in the range of 395–404 K for the same aging time (t_a_) 30 min (the main part of figure). This experiment was performed in order to establish the aging temperature, T_a_, for which the enthalpy relaxation value becomes maximum. The results show that ∆H_a_^max^ was observed at T_a_ = 400.15 K as is pointed in the insert of Figure 5.

**Figure 6:**
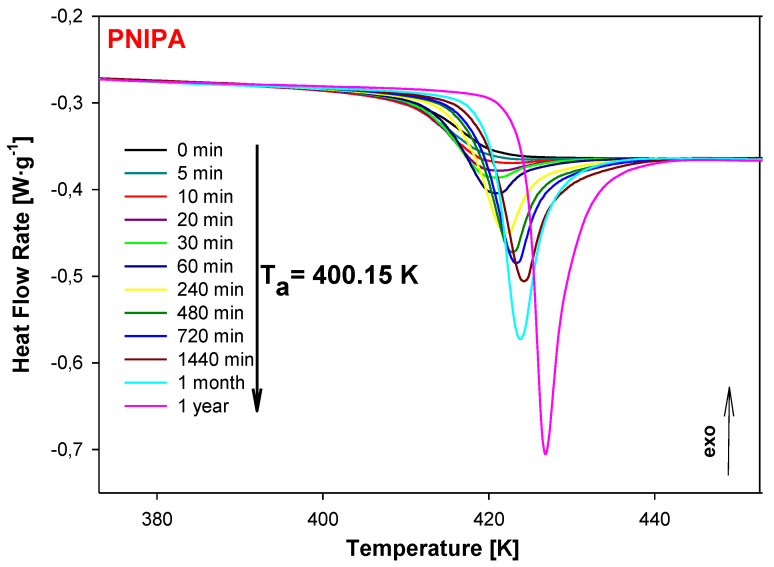
The change of heat flow rate of unaged (t_a_ = 0 min) and aged at T_a_ = 400.15 K of PNIPA versus temperature. The aging time of PNIPA was used in the range of 5 min to 1 year.

Next, the sample of PNIPA was aged at 400.15 K for variable times from 5 min to 1 year and the results of the heat flow rate versus temperature during the heating at 10 K/min were presented in Figure 6. For comparison, also in Figure 6, the unaged data of PNIPA (t_a_ = 0 min) is shown. The growth of endothermic peaks of the enthalpy relaxation was observed during the applied longer aging time.

**Figure 7:**
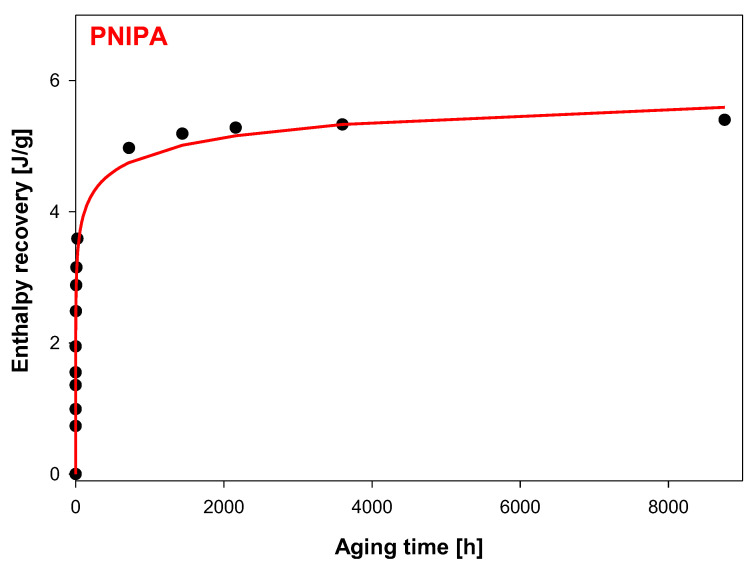
Enthalpy recovery vs. aging time at T_a_ = 400.15 K for the amorphous PNIPA. The solid line is the result of fit the experimental data to Kohlrausch–Williams–Watts (KWW) function (R^2^ = 0.9894).

The enthalpy recovery from endothermic peaks (Figure 7) was estimated based on the difference of areas appointed between curves of heat flow rates of the aged and unaged PNIPA (∆H_a_ = B–A) according to Figure 2. The results of ∆H_a_ = f(t_a_) for the aging temperature at T_a_ = 400.15 K from the aging time 5 min to 1 year are presented in Table 1.

**Figure 8:**
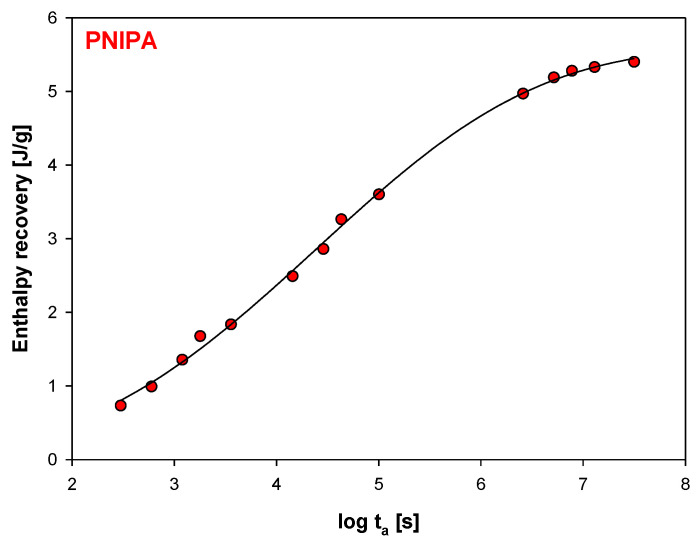
Changes of enthalpy recovery versus logarithmic function of aging time (R^2^ = 0.9984).

In addition, Figure 7 shows the graphic dependence of the enthalpy recovery (∆H_a_) on the aging time (t_a_) and Figure 8, versus logarithmic aging time (log t_a_). The results indicate on the significant change of the enthalpy relaxation in the range of 10 min to 1 month. Subsequently, a leisurely (or small) change of ∆H_a_ was observed to achieve a plateau state.

The experimental data (black dots) from Figure 7 were fitted to the Kohlrausch–Williams–Watts (KWW) function using the following equation [64,65]:

(3) $$∆H_{a}=∆H_{\inf}{\left[1-e^{-{\left(\frac{t_{a}}{\tau }\right)}^{\beta }}\right]}^{\prime }$$

where: ∆H_a_–the enthalpy relaxation, ∆H_inf_–the equilibrium enthalpy relaxation, t_a_–the aging time, τ–the relaxation time, and β–the exponential parameter [54,63].

The solid line in Figure 7 is the calculated enthalpy relaxation obtained with parameters β = 0.18, and τ = 208.74 h from fitting to the KWW Equation (3) and with the equilibrium enthalpy recovery, ΔH_inf_ estimated based on Equation (1), and equals 6.61 J/g. Figure 8 shows changes of enthalpy recovery versus logarithmic function of aging time obtained based on Figure 7.

**Figure 9:**
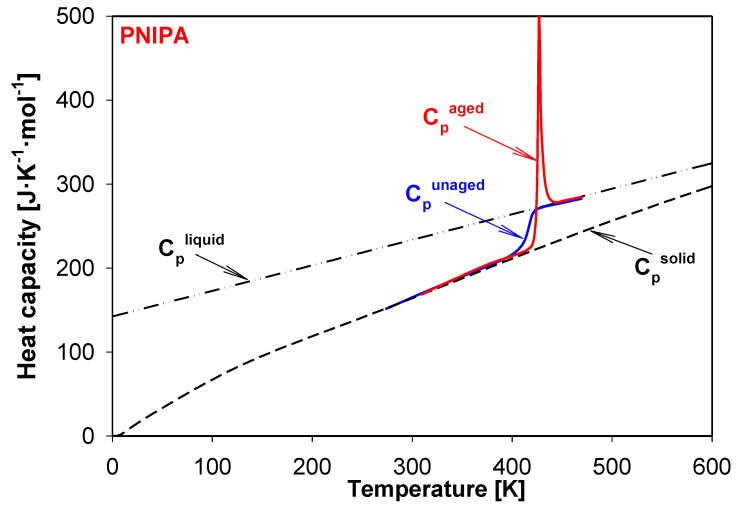
Experimental apparent heat capacities of aged and unaged PNIPA versus temperature were compared with equilibrium solid (C_p_^solid^) and liquid (C_p_^liquid^) heat capacities. The result of physical aging process was obtained based on the storage of material at 400.15 K for 1 year.

In Figure 9, the experimental apparent heat capacities for the aged and unaged of amorphous PNIPA were shown. The data were obtained during the heating with the rate of 10 K/min. The example of aging PNIPA presents the result obtained during the isothermal aging within 1 year at 400.15 K. The experimental results for both unaged and aged PNIPA were presented in the reference to equilibrium heat capacities at the solid (C_p_^solid^) and liquid (C_p_^liquid^) states of PNIPA. Temperature of glass transition was estimated as 415 K based on the heat capacity measurement of the unaged PNIPA by the standard DSC. Based on earlier studies [54], the glass transition temperature (T_g_) of the unaged PNIPA was estimated as 415 K, and the change of heat capacity, ΔC_p_, at T_g_ (415 K) was found as 50.36 J·mol^−1^·K^−1^ using equilibrium baselines for solid and liquid states of PNIPA. Based on these (ΔC_p_, T_g_, and T_a_) data, the equilibrium enthalpy relaxation was estimated as ∆H_inf_ = 747.85 J·mol^−1^ (6.61 J/g) using Equation (1).

**Figure 10:**
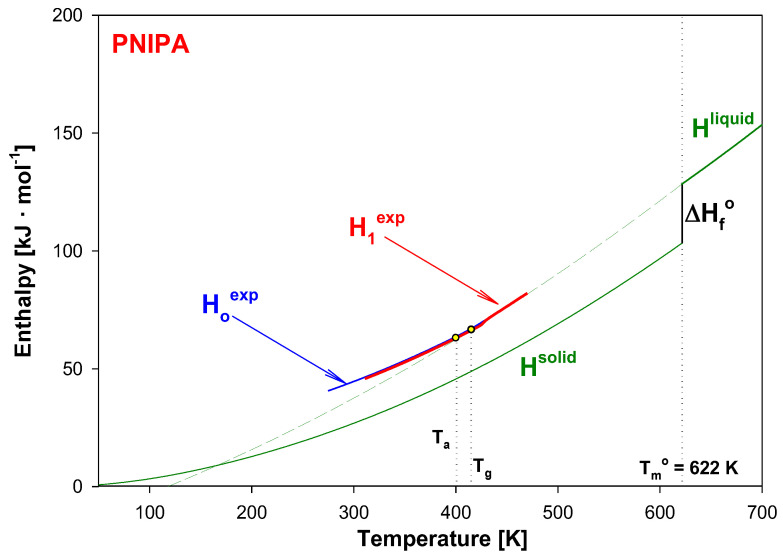
Experimental enthalpy of aged (H_1_^exp^) and unaged (H_o_^exp^) of PNIPA versus temperature in the frame of equilibrium enthalpy of solid (H^solid^) and liquid (H^liquid^). Analysis is presented for sample of poly(*N*-isopropylacrylamide) isothermally aged at 400.15 K for 1 year.

**Figure 11:**
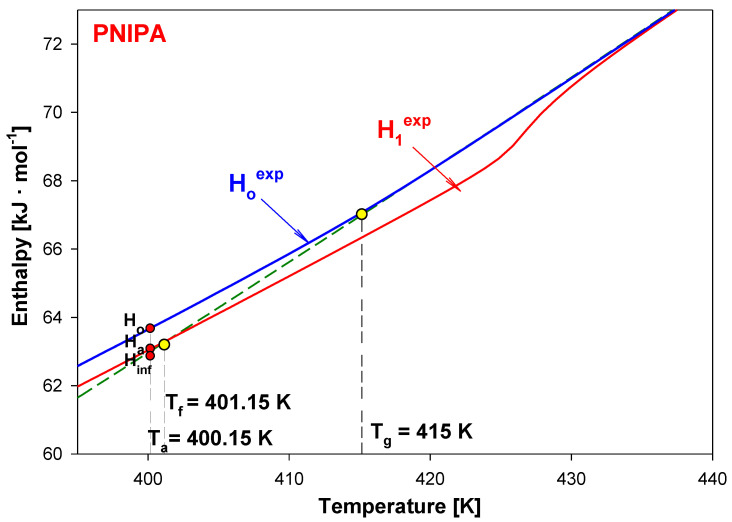
Enlargement of the experimental enthalpy of material subjected to aging for one year (H_1_^exp^) and for unaged PNIPA (H_o_^exp^) from Figure 10 in the region of the glass transition.

Knowing heat capacities and parameters of transitions, the total enthalpy was calculated for all data presented in Figure 9. Figure 10 shows these total enthalpies in range of 0 to 700 K, and Figure 11 presents the enlargement of those enthalpies in the glass transition region from 395 K to 435 K. The structural recovery process of PNIPA after 1 year annealing (t_a_ = 1 year) at 400.15 K was presented. Figure 10 and Figure 11 show the integral enthalpy function of aged (H_1_^exp^) after 1 year at 400.15 K and unaged (H_o_^exp^) PNIPA versus temperature, in the glass transition region, and in Figure 11 compared with equilibrium liquid (H^liquid^) and solid enthalpy (H^solid^). The dash line shows the extension of equilibrium enthalpy at liquid state (H^liquid^) towards a low temperature. Based on intersection of the dash line (H^liquid^) and experimental enthalpy of aged PNIPA (H_1_^exp^), the fictive temperature of T_f_ = 401.15 K was determined. In Figure 10, the solid enthalpy, (H^solid^) in the melting temperature at T_m_° = 622 K increased about a value of heat of fusion (∆H_f_°) to reach the level of the liquid enthalpy (H^liquid^).

In the case of the unaged material, the experimental enthalpy of PNIPA (H_o_^exp^) changes the curve slope at the temperature of glass transition (T_g_ = 415 K). The enthalpy recovery of aged PNIPA (ΔH_a_) within 1 year was estimated at the aging temperature (T_a_ = 400.15 K) based on difference of enthalpies between H_a_ and H_o_ points, as it is in Figure 11 where the value of ΔH_a_ equals 609 J/mol. The difference between H_a_ and H_inf_ points equals the value of equilibrium enthalpy recovery (∆H_inf_) and gives a value 747 J·mol^−1^, which agrees with the value that was calculated earlier from Equation (1).

Using the above data, the recovery parameter (φ) was also calculated based on the Equation (2). In the case of PNIPA after t_a_ = 1 year, the recovery parameter equals 0.18.

**Figure 12:**
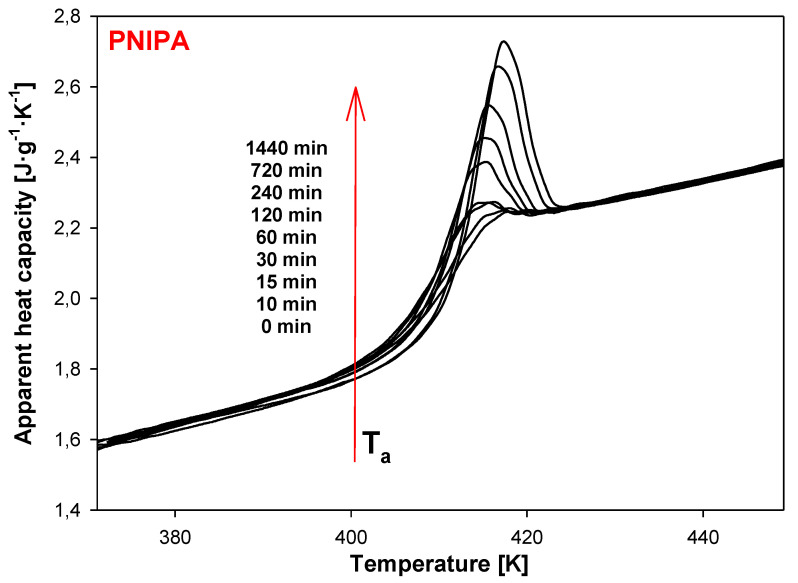
Isothermal aging of poly(*N*-isopropylacrylamide) at the aging temperature, T_a_ = 400.15 K after different aging time, 0–1440 min obtained based on temperature-modulated differential scanning calorimetry (TMDSC) (underlying heating rate: *q* = 3 K/min, modulation amplitude: A = 1.5 K, and modulation period: p = 60 s).

In order to separate changes of heat capacity (ΔC_p_) from enthalpy relaxation (ΔH_a_) in the glass transition region, the temperature-modulated differential scanning calorimetry (TMDSC) was applied. In this TMDSC method, the amorphous PNIPA was aging at isothermal conditions at the aging temperature, T_a_ = 400.15 K the same as in standard DSC method, and in the aging time range of 0 to 1440 min (24 h). Results were observed on the underlying heating scans at 3 K/min, modulation amplitude A = 1.5 K, and modulation period, p = 60 s. Figure 12 shows the results of total, apparent heat capacity from TMDSC versus temperature in the glass transition temperature region of PNIPA after isothermal aging at T_a_ = 400.15 K. The bigger endothermic peaks of the enthalpy of relaxation overlapped on the changes of heat capacity were observed for a longer aging time. Figure 12 presents also the total apparent heat capacities of unaged PNIPA measured by TMDSC.

**Figure 13:**
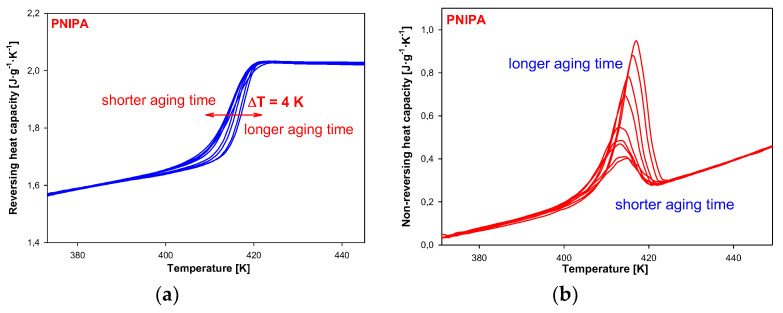
(**a**) Reversing heat capacity of amorphous PNIPA versus temperature obtained based on the isothermal aging. (**b**) Non-reversing heat capacity of amorphous PNIPA versus temperature obtained based on the isothermal aging.

Figure 13 shows the results of reversing and non-reversing heat capacities versus temperature, obtained from total C_p_ after Fourier’s analysis. The dependence of reversing heat capacity on temperature (Figure 13a) shows changes of heat capacity for a different aging time in the range of 0 to 1440 min (24 h). The higher values of glass transition temperature were observed for a longer aging time. These data are listed in Table 2. Total difference between the glass transition temperature of unaged and aged reversing heat capacity for 1440 min (24 h) was estimated around 4 K. Figure 13b presents non-reversing heat capacities versus temperature. The results show that bigger endotherm peaks of enthalpy relaxation for a longer aging time of PNIPA.

Estimation of enthalpy recovery values based on these non-reversing heat capacities was not made due to limitation of the Fourier’s analysis as it was presented by Simon and co-workers in Ref. [66].

### 2.2. Non-Isothermal Aging of PNIPA

**Figure 14:**
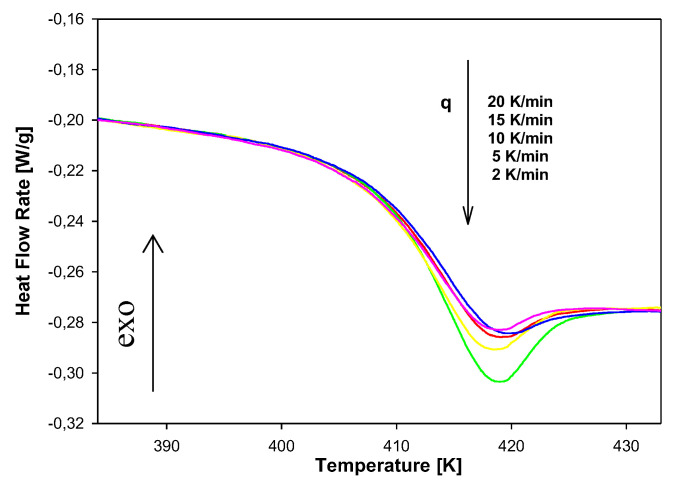
The dependence of heat flow rate on temperature obtained after the non-isothermal aging of PNIPA with a different cooling rate in the range of 2 K/min to 20 K/min.

Figure 14 presents results of heat-flow rates versus temperature on heating scans at 10 K/min for the non-isothermal aging of amorphous PNIPA after the cooling process from 473 K to 363 K with different cooling rates from 2 to 20 K/min. Data show that for a lower cooling rate, the bigger endotherm peaks are observed. These peaks overlap on a change of heat capacity at the T_g_ region.

Similar to the isothermal physical aging, the enthalpy recoveries for the non-isothermal aging process were estimated. Results are presented in Table 3.

**Figure 15:**
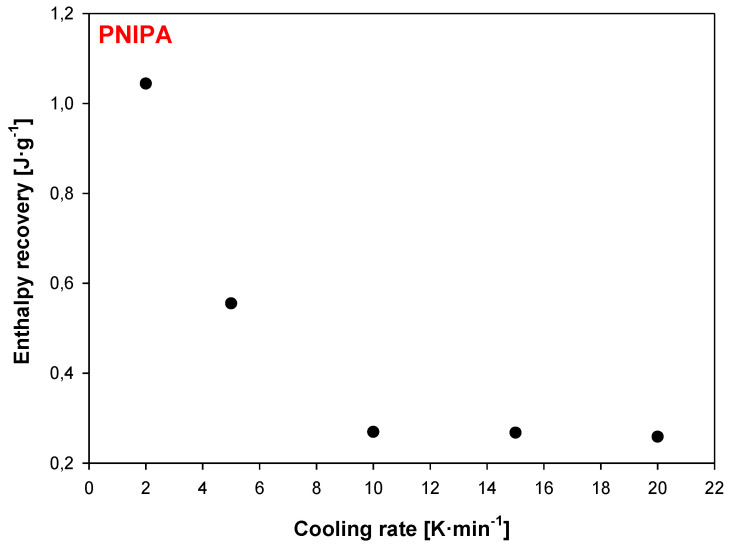
Enthalpy recovery versus different cooling rates obtained after the non-isothermal aging of amorphous PNIPA.

Figure 15 shows the graphic dependence of enthalpy recovery (∆H_a_) on cooling rates (*q*). The results indicate on the significant change of enthalpy relaxation in the range of cooling rate 2 K/min to 10 K/min. Subsequently, relevant changes of ∆H_a_ were not observed using q above 10 K/min.

**Figure 16:**
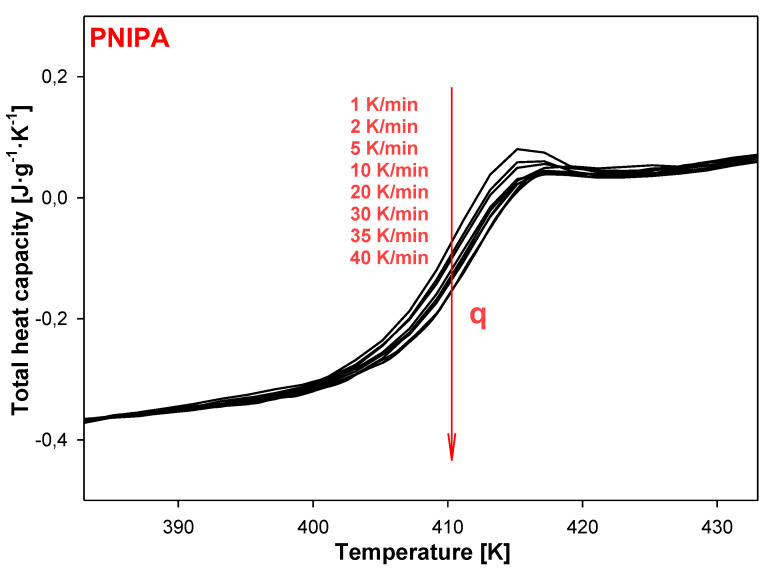
Non-isothermal aging of poly(*N*-isopropylacrylamide) with a different thermal history obtained based on variety of cooling rates in range of 1 K/min to 40 K/min. Total heat capacity was obtained based on heating scans by the temperature-modulated differential scanning calorimetry, TMDSC.

**Figure 17:**
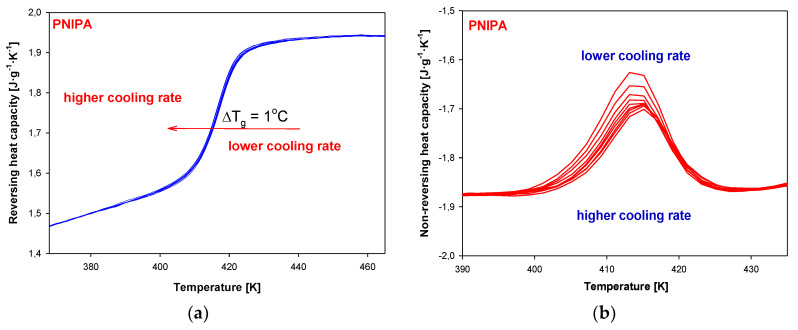
(**a**) Reversing heat capacity of amorphous PNIPA versus temperature obtained based on the non-isothermal aging. (**b**) Non-reversing heat capacity of amorphous PNIPA versus temperature obtained based on the non-isothermal aging.

Figure 16 presents the change of total heat capacities versus temperature in the glass temperature region of PNIPA obtained from TMDSC after the non-isothermal aging. The bigger endotherm peaks of the enthalpy of relaxation overlapped on the changes of heat capacity were obtained using a lower cooling rate (Figure 17a). Application of the higher cooling rates causes change of the glass transition temperature about 1 K in reference to use of *q* = 1 K/min (Figure 17b).

## 3. Materials and Methods

Poly(*N*-isopropylacrylamide) was prepared using free radical polymerisation in water as was discussed in Refs. [54,63,67,68]. The PNIPA used in this paper is this same sample a that in Ref. [54]. It is an amorphous substance and molar mass of its repeating unit equals 113.16 g/mol. The earlier study described in Reference [54] indicates that this PNIPA is a linear polymer. In addition, according to Ref. [54], the weight average molar weight, M_w,_ equals 731,800 g/mol and the number average molar weight of M_n_ = 343,246 g/mol.

In order to carry out measurements of thermal properties of aged and unaged PNIPA standard DSC and temperature-modulated differential scanning calorimetry (TMDSC) of a Q1000^TM^ (TA Instruments) were used in the temperature range of 183–473 K. The all effects on the DSC curves was shown as exo up. This heat-flux calorimeter was linked with a mechanical refrigerator for controlling cooling/heating process of the sample. All experiments were performed in a nitrogen atmosphere with a constant flow rate of around 50 mL/min.

**Figure 18:**
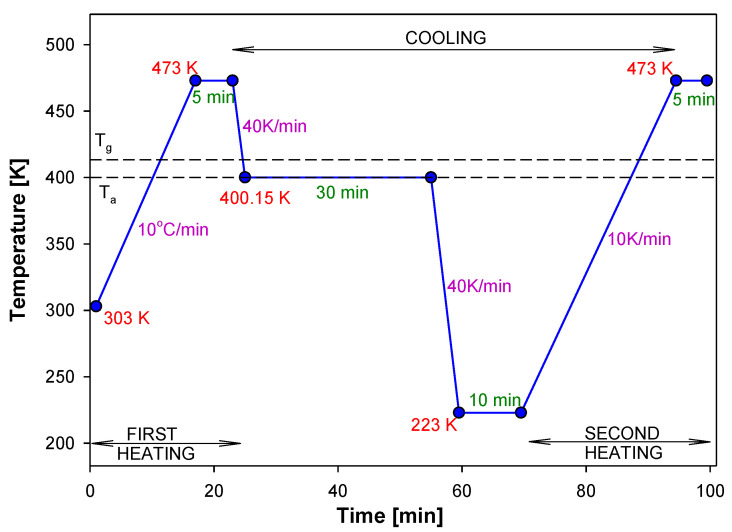
Example of the isothermal aging program during a measurement by DSC.

The experimental apparent heat capacities were obtained by standard DSC from the evaluation of measured heat-flow rates at a heating rate of 10 K/min after previous cooling at 10 K/min. The samples of PNIPA were aged isothermally at T_a_ = 400.15 K for different aging times. During experiments, samples were first preheated above T_g_ temperature for PNIPA to 473 K to erase a thermal history, and then the samples were cooled below T_g_ and aged for a given time. Next, the samples were cooled and reheated at 10 K/min from 223 K to 473 K to obtain scan for the aged substance. Figure 18 shows an example of temperature program versus time for monitoring the structural recovery process of PNIPA.

In order to perform a non-isothermal physical aging process of amorphous PNIPA, the samples were first heated above the T_g_ temperature to 473 K then cooled with different cooling rate from 1 to 40 K/min until below T_g_ to 363 K. Finally, the reheating at 1 K/min from 303 K to 473 K was performed to obtain results of the heat flow rate for aged PNIPA. Such measurement was repeated for the each cooling rate.

Calibration of the temperature and the heat flow rate in the DSC apparatus was performed using the onset melting temperature, T_m_(onset) = 429.6 K (156.6 °C), and the heat of fusion ΔH_f_ = 28.45 J/g (3.281 kJ/mol) of indium. In order to obtain accurate results of C_p_, the heat capacity was calibrated with a sapphire. For calibrated heat capacity data, three measurements were performed: a blank/reference, a calibration with sapphire, and the examinee sample. On this basis, the heat capacity, at a steady state, was determined from the following equation [69,70]:

(4) $$m\cdot C_{p}=K\frac{∆T}{q}+C_{s}\frac{d∆T}{{\mathrm{dT}}_{s}},$$

where K is Newton’s constant, ΔT is the temperature difference between reference and sample, C_s_ is the heat capacity of the sample calorimeter including sample and aluminum pan, T_s_ is the temperature of the sample, and q is the heating rate [71]. The masses of the samples used for measurements were 10–30 mg. The heat capacity data were collected from the second heating run after controlled cooling or from the run after isothermal aging. The experiments were repeated for other aged and unaged scans. The accuracy of the C_p_ measurements is estimated to be ±3% or better. From obtained results, the enthalpy relaxation (∆H_a_) was determined from the difference in the area under aged and unaged scans, as shown in Figure 2 [27,45].

In addition, using TMDSC method, the isothermal aging of amorphous PNIPA was carried out in range of 183–473 K with the following parameters of measurement: modulation amplitude, A = 1.5 K, underlying heating rate of *q* = 3 K/min, and modulation period, p = 60 s. In the case of non-isothermal aging of amorphous PNIPA, the TMDSC measurement was performed with a different thermal history (after different cooling rates: 2 K/min–40 K/min) in range of 363 K–473 K using A = 0.5 K, *q* = 1 K/min, and p = 60 s.

## 4. Conclusions

The structural recovery process of poly(*N*-isopropylacrylamide) was analyzed for the first time using advanced thermal analysis with the application of heat capacity and thermodynamic functions of total enthalpy. The measurements were carried out at the aging temperature of 400.15 K (about 15 K below the temperature of glass transition) by DSC. The equilibrium solid and liquid enthalpies were used to show and estimate the enthalpy recovery of PNIPA. The results were obtained based on aging in isothermal conditions in range of 5 min to 1 year and non-isothermal conditions using different cooling rates of full amorphous PNIPA. The KWW (Kohlrausch–Williams–Watts) model was applied to determine the kinetics of the aging process of PNIPA. From the best fit, the KWW parameters, the relaxation time *τ* = 208.74 h and the coefficient β = 0.18 describing the distribution of relaxation times were obtained during the isothermal physical aging process. With a stretching exponent β between 0 and 1, our result of exponential constant β value is rather small and the distribution of *τ* states is wide. The progress of physical aging process in isothermal conditions was also described using the recovery parameter. It was established that, after aging by 1 year at 400.15 K, the recovery parameter was φ = 0.18. The low value of the recovery parameter points to a high level of a relaxation of material in the reference to the equilibrium state. In the equilibrium state, where φ = 0, the enthalpy recovery achieves the value of equilibrium enthalpy recovery. The obtained value of the φ parameter suggests a state near an aging plateau. The estimated enthalpy recovery based on the (∆H_a_ = area B-area A) area scheme agreed with the value obtained from advanced thermal analysis using the total enthalpy presentation. The value of fictive temperature (= 401.15 K) was obtained based on the intersection of the liquid enthalpy and experimental enthalpy of aged PNIPA. Many experiments [58,59,60,61,62] obtained that the extrapolated liquid line is not reached even at the completion of enthalpy recovery. Noteworthy is the fact that the value of T_f_ for the aged PNIPA by 1 year is near the aging temperature (T_a_ = 400.15 K). The difference between both was only 1 K, which means that amorphous PNIPA almost reaches the equilibrium state.

## Figures and Tables

**Table 1:** The change of enthalpy recovery (∆H_a_) of PNIPA versus the different aging time (t_a_).

Aging Time (T_a_)	Enthalpy Recovery (∆H_a_) [J/G]	Enthalpy Recovery (∆H_a_) [J/Mol]
5 min	0.73	82.61
10 min	0.99	112.03
20 min	1.36	153.90
30 min	1.55	190.11
60 min	1.95	208.21
240 min	2.48	281.77
480 min	2.88	323.64
720 min	3.15	368.90
1 day	3.59	407.38
1 month	4.97	562.41
2 months	5.19	587.30
3 months	5.28	597.48
5 months	5.33	603.14
12 months	5.40	611.06

**Table 2:** Dependence of glass transition temperature on an aging time of the amorphous poly(*N*-isopropylacrylamide) obtained based on reversing heat capacity under the isothermal aging process measured by TMDSC (see Figure 13a).

Temperature of Glass Transition [K]	Aging Time [Min]
413.50	0
414.54	10
414.55	15
414.56	30
415.05	60
415.55	120
415.94	240
416.92	720
417.75	1440

**Table 3:** The enthalpy recovery (∆H_a_) of PNIPA versus the different thermal history, cooling rate (q).

Cooling Rate, Q [K/Min]	Enthalpy Recovery, ∆H_a_ [J/G]
2	1.0440
5	0.5552
10	0.2692
15	0.2675
20	0.2585
